# Regulation of tamoxifen sensitivity by the PLAC8/MAPK pathway axis is antagonized by curcumin-induced protein stability change

**DOI:** 10.1007/s00109-021-02047-5

**Published:** 2021-02-21

**Authors:** Misha Mao, Dengdi Hu, Jingjing Yang, Yongxia Chen, Xun Zhang, Jianguo Shen, Rongyue Teng, Jichun Zhou, Linbo Wang

**Affiliations:** 1 Department of Surgical Oncology, Affiliated Sir Run Run Shaw Hospital, Zhejiang University School of Medicine, Hangzhou, China; 2 Department of Hepatobiliary Surgery, Cixi People’s Hospital of Zhejiang Province, Ningbo, China

**Keywords:** Breast cancer,, PLAC8,, Tamoxifen resistance,, MAPK/ERK pathway,, Curcumin,, Ubiquitination

## Abstract

Tamoxifen resistance remains the major obstacle to the estrogen receptor positive breast cancer endocrine therapy. Placenta-specific 8 (PLAC8) has been implicated in epithelial-mesenchymal transition and tumorigenesis. However, the molecular mechanisms underlying PLAC8 function in the context of tamoxifen resistance are unclear. Curcumin has attracted considerable attention in the last decades. It is isolated from *Curcuma longa* and has beneficial effects in cancer therapy. We studied this property by using MCF-7 and tamoxifen-resistant breast cancer cells (MCF-7/TAM) cell lines. PLAC8 can regulate MCF-7/TAM cell drug sensitivity through the MAPK/ERK pathway and shows the potential effects of curcumin or as a possible druggable target against tamoxifen failure.

**Supplementary Information:**

The online version contains supplementary material available at 10.1007/s00109-021-02047-5.

## Introduction

Breast cancer (BC) is the most common malignant tumor and the second leading cause of cancer-related death in women [1, 2]. It is a heterogeneous disease that can be classified into multiple subtypes, each with distinctive histological and biological features. The most common subtype is the hormone receptor-positive breast cancer and approximately 70–75% of all breast cancers expressing the estrogen receptor (ER) and/or progesterone receptor (PR) [3]. The use of endocrine therapy to block ER activity is an important treatment for these patients. Tamoxifen, a selective ER modulator, is the most frequently used drug for the management of ER positive breast cancer [4]. However, its de novo or acquired resistance limits the efficacy of tamoxifen. The aberrant activated factors or pathway contribute to tamoxifen resistance, such as ER mutation and the activation of PI3K/AKT pathway [5, 6]. An alternative or supplementary therapeutic strategy is needed to effectively treat tamoxifen-resistant patients.

PLAC8 was found to be highly expressed in the mouse placenta [7]. Accumulating evidence have shown that PLAC8 is involved in the participation of cancer processes, including in the hepatocellular carcinoma, nasopharyngeal carcinoma and lung cancer [8–10]. PLAC8 has a pivotal oncogenic or tumor suppressor role in cancer progression. We have confirmed that PLAC8 can suppress breast cancer apoptosis by activating the PI3k/AKT/NF-κB pathway [11]. However, whether PLAC8 is involved in tamoxifen resistance in breast cancer is still unclear. Curcumin, a major component of the rhizome of *Curcuma longa*, interacts with various proteins and regulates their expression and activity, including PI3K/Akt, NF-kB, and c-Myc [12–14]. Curcumin influences the proliferation of breast cancer cells and other types of cancer cells, such as gastric cancer cell, esophageal squamous cell carcinoma, and prostate cancer cells [13, 15, 16]. Curcumin may be an promising drug in breast cancer treatment.

In this study, we aimed to evaluate the effect of PLAC8 on the viability of tamoxifen resistant breast cancer cells. We further determined whether PLAC8 regulated MCF-7/TAM proliferation, migration, and invasion through the MAPK/ERK pathway. In addition, we assessed whether curcumin could induce the ubiquitination of PLAC8. Our work demonstrates that PLCA8 can be a therapeutic target for tamoxifen-resistant patients and curcumin as a new promising drug for reversing tamoxifen-resistance via targeting PLAC8/MAPK pathway in a ubiquitination dependent way.

## Method and materials

### Tissue specimens

Twelve samples of tamoxifen-sensitive and thirteen samples of tamoxifen-resistant breast cancer tissues which including their breast cancer metastatic tissues were obtained from patients who had been histopathologically and clinically diagnosed from 2002 to 2015 in the Affiliated Sir Run Run Shaw Hospital, Zhejiang University School of Medicine. Written informed consent was obtained from each patient. In each case, protein expression was evaluated by two pathologists (double blinded). The staining for PLAC8 was divided into four scores: strong, +3; moderate, +2; weak, +1; and negative, 0. Specimens with scores of +3 or +2 were defined as having high expression, and those with scores of +1 or 0 were defined as having low expression. The study was approved by the Ethics Committee of Affiliated Sir Run Run Shaw Hospital, Zhejiang University School of Medicine.

### Cell lines and culture

MCF-7 cell lines were purchased from the American Type Culture Collection (ATCC) and maintained in RPMI 1640 Medium (21870092, Thermo Scientific) supplemented with 10% foetal bovine serum (16000044, Gibco) and 5% glutamine. According to the methodology reported, tamoxifen-resistant MCF-7 (MCF-7/TAM) sublines were established by prolonged growth of MCF-7 cells in 1μM tamoxifen media over 6 months [17–19]. The characteristics of tamoxifen-resistant breast cancer cell lines were shown in Supplementary Figure 1. The cell lines grew in a humid atmosphere containing 5% CO_2_ at 37 °C.

### Drugs and inhibitors

Tamoxifen (T5648) and curcumin (C1386) were purchased from Sigma-Aldrich and were soluble in a DMSO in vitro assay. Tamoxifen and curcumin were soluble in 10% DMSO, 50% PEG 300, and distilled water in the in vivo assay. MG-132 (S2619), ERK inhibitor SCH772984 (S7101), and P38 inhibitor SB2020190 (S1077) were purchased from Selleck and were soluble in DMSO in vitro.

### Scanning electron microscopy

Cells were fixed with 2.5% glutaraldehyde for 1 h at room temperature and post-fixed overnight at 4 °C. The samples were washed thrice with PBS. Samples were dehydrated by the increasing ethanol gradient and embedded in epoxy resin. Samples were observed under a scanning electron microscope (Nova Nano 450, Thermo Fisher Scientific, Inc).

### Transfection

Three short interfering RNAs targeting PLAC8 (Si-PLAC8#1, Si-PLAC8#2,Si-PLAC8#3) and a scrambled control siRNA (Si-NC)were designed and purchased from RiboBio (Guangzhou, China). The Si-PLAC8 sequences were CCTTGGGTGTCAAGTAFCA(Si-PLAC8#1), GGAACAAGCGTCGCAATGA(Si-PLAC8#2), and GGAGAGCCATGCGTACTTT(Si-PLAC8#3). PLAC8 cDNA and negative control cDNA were subcloned into pcDNA3.1. Cells were transfected with siRNAs or plasmids using Lipofectamine 3000 (Invitrogen, USA) according to the manufacturer's instructions.

### Immunofluorescence staining

Cells were briefly seeded onto glass coverslips in 24-well plates up to 50–60% confluence. Cells were washed thrice and blocked in PBST (PBS containing 0.1% Tween) containing 2.5% bovine serum albumin (Sigma-Aldrich, St Louis, USA) at room temperature for 30 min. Cells were washed thrice and then incubated with PLAC8 antibody (1:200) at 37 °C for 1 h followed by Alexa 488-conjugated (green) goat anti-rabbit antibody (1:1000) (Multisciences, Hangzhou, China) to detect the target protein. DAPI was used to visualize the cell nuclei. Images were acquired using a Nikon laser scanning confocal microscope (Nikon Instruments Inc., Melville, NY, USA).

### Western blot analysis

Cells were lysed with RIPA lysis buffer supplemented with 1× PMSF. Whole-cell lysates were separated by SDS-PAGE (BioRad, Berkeley, CA, USA) and transferred to PVDF membranes (Millipore, Billerica, MA, USA). Then, the blots were blocked with 5% non-fat milk for 1 h. The primary antibodies used in this study included PLAC8 (#13885, 1:500), E-cadherin (#3195, 1:1000), N-cadherin (#2348, 1:1000), vimentin (#5741, 1:1000), ubiquitin (#3933, 1:1000), P38 (#8690, 1:1000), p-P38 (#4511, 1:1000), ERK1/2 (#4695, 1:1000), and p-ERK1/2 (#9010, 1:1000). These antibodies were purchased from Cell Signaling Technology (New England Biolabs, Herts, UK). GAPDH (1:1000) antibodies were purchased from Santa Cruz Biotechnology (CA, USA). The blots were probed with primary antibody overnight at 4 °C and then incubated with the secondary antibody (Abcam, Cambridge, MA, USA) for 1 h at room temperature. Reactive bands were visualized with ECL Plus reagents by using LAS-4000 mini.

### Cell proliferation assay

Cells (0.5 × 10^4^) were seeded onto a 96-well culture plate for 12, 24, or 72 h with or without the drug. Cell viability was evaluated using the MTT assay (Cell Titer 961 AQueous One Solution Cell Proliferation Assay, Promega). The absorbance was measured at 490 nm by using a BioTek ELx800 absorbance microplate reader.

### Wound-healing assay

Cells (5 × 10^5^) were seeded onto six-well plates and incubated up to 80–90% cell confluence. Scratch wounds were made using the pipette tip. The cells were washed thrice with PBS to remove cell debris and then replaced with complete medium. The scratch was recorded under a phase contrast microscope at the time of wound generation and at 0, 24, and 48 h. The gap widths were measured using ImageJ.

### Transwell migration and invasion assay

For the Transwell (Corning Costar, Cambridge, MA, USA) migration assays, cells were collected in the medium without serum. Cell invasion was measured using Transwell chambers with Matrigel (Corning Costar, Cambridge, MA, USA). Cells (5 × 10^4^) in 100 μl of medium without serum were transferred into the upper chamber of the Transwell, and 600 μL of medium containing 10% FBS was added to the lower chamber. After incubation for 24 h, cells on the upper surface of the membrane were carefully removed using a cotton swab. The membrane was fixed with 4% paraformaldehyde and stained with 0.5% crystal violet solution for 15 min. Images were captured under a microscope (zeiss, Primovert).

### RNA isolation and quantitative real-time PCR

Total RNA was extracted from cells and tissues using TRIzol (Invitrogen, Carlsbad, CA) reagent according to the manufacturer’s instructions. Total cDNA was synthesized using the HifiScript cDNA synthesis kit (Cwbio, Jiangsu, China). Quantitative real-time PCR was performed using the UltraSYBR mixture (CW0957, Cwbio, Jiangsu, China) in ABI 7300 (Applied Biosystems Inc., USA). The reactions were carried out under the following conditions: 95 °C for 10 min; 40 cycles of 95 °C for 15 s, 60 °C for 1 min; and followed by 95 °C for 15 s, 60 °C for 1 min, 95 °C for 15 s, and 60 °C for 15 s. The mRNA expressions were assessed by evaluating the threshold cycle (CT) values. GAPDH mRNA was used as an endogenous control for mRNAs. Relative expression was calculated using the relative quantification equation (RQ) = 2−ΔΔCt. Primer blasting and the melting curve were analyzed to ensure the specificity of amplification. All samples were amplified thrice by real-time, and the expression was normalized to GAPDH. The following primer sequences were used:

- GAPDH forward 5′-TGACTTCAACAGCGACACCCA-3′
- GAPDH reverse 5′-CACCCTGTTGCTGTAGCCAAA-3′
- PLAC8 forward 5′-GGAACAAGCGTCGCAATGAG-3′
- PLAC8 reverse 5′-AAAGTACGCATGGCTCTCCTT-3′
- N-cadherin forward 5′- AGCTCCATTCCGACTTAGACA -3′
- N-cadherin reverse 5′- CAGCCTGAGCACGAAGAGTG-3′
- E-cadherin forward 5′- AAAGGCCCATTTCCTAAAAACCT-3′
- E-cadherin reverse 5′- TGCGTTCTCTATCCAGAGGCT-3′
- Vimentin forward 5′- AGTCCACTGAGTACCGGAGAC -3′
- Vimentin reverse 5′- CATTTCACGCATCTGGCGTTC-3′.

### Tumor xenografts in nude mice

Twenty-four BALB/c nude mice (4 weeks old, from Shanghai Laboratory Animal Center, Shanghai, China) were housed in a specific pathogen-free environment. A total of 2 × 10^6^ MCF-7/TAM cells in 100 μl of PBS with 100 μl of growth factor-reduced basement membrane matrix (Corning Costar, Cambridge, MA, USA) were injected into the right sub-axillary region of each mouse. The tumor size was measured twice per week using a slide caliper, and the tumor volume was calculated using the formula: 0.5 × *A* × *B*^*2*^, where *A* is the length of the tumor, and *B* is the width of the tumor [20]. Therapeutic experiments started when the tumor reached approximately 100 mm^3^ after approximately 14 days. Mice were randomly divided into the four following groups (*n* = 6/group): control (vehicle), tamoxifen (15 mg/kg every 3 days, intraperitoneal injection), curcumin (30 mg/kg every 3 days, intraperitoneal injection), and tamoxifen (15 mg/kg every 3 days, intraperitoneal injection) plus curcumin (30 mg/kg every 3 days, intraperitoneal injection). Then, 24 days after drug injection, the mice were euthanized, and the subcutaneous growth of each tumor was examined. Wet tumor weight was expressed as mean weight ± standard deviation (SD) in each group. Some tumor tissues were fixed with 10% paraformaldehyde for immunohistochemical analysis. Other tumor tissues were frozen immediately in liquid nitrogen for Western blot analysis. This study was approved by the Ethics Committee for Animal Studies of Zhejiang University (Hangzhou, China).

### Immunohistochemical staining

The slices of paraffin-embedded tissues were deparaffinized and rehydrated in xylene and graded alcohol solutions and then blocked with 3% H_2_O_2_ for 5 min and 3% bovine serum albumin (Roche, Hong Kong, China) for 15 min. The slices were stained with PLAC8 (1:200) and Ki-67 (1:500) (100130-MM22, Sino Biological, Beijing, China) for 1 h at 37 °C. The tissues were washed thrice with PBS for 3 min and then stained with the secondary antibody from the GT Vision III immunohistochemical assay kit (GK500710, Gene Tech, Shanghai, China) according to the manufacturer’s instructions. All images were captured using a fluorescence microscope (Olympus BX-51, Japan).

### Statistical analysis

The comparisons between multiple groups were performed using multiple comparisons by one-way ANOVA. Comparisons between groups were performed using Student’s *t*-test. All data were obtained from at least three independent experiments. The values are presented as the mean ± SD (*, *p* < 0.05; **, *p* < 0.01; NS, not significant). All analyses were performed in GraphPad Prism 7.0 (San Diego, CA, USA).

## Results

### PLAC8 upregulation is associated with tamoxifen resistance

**Fig. 1:**
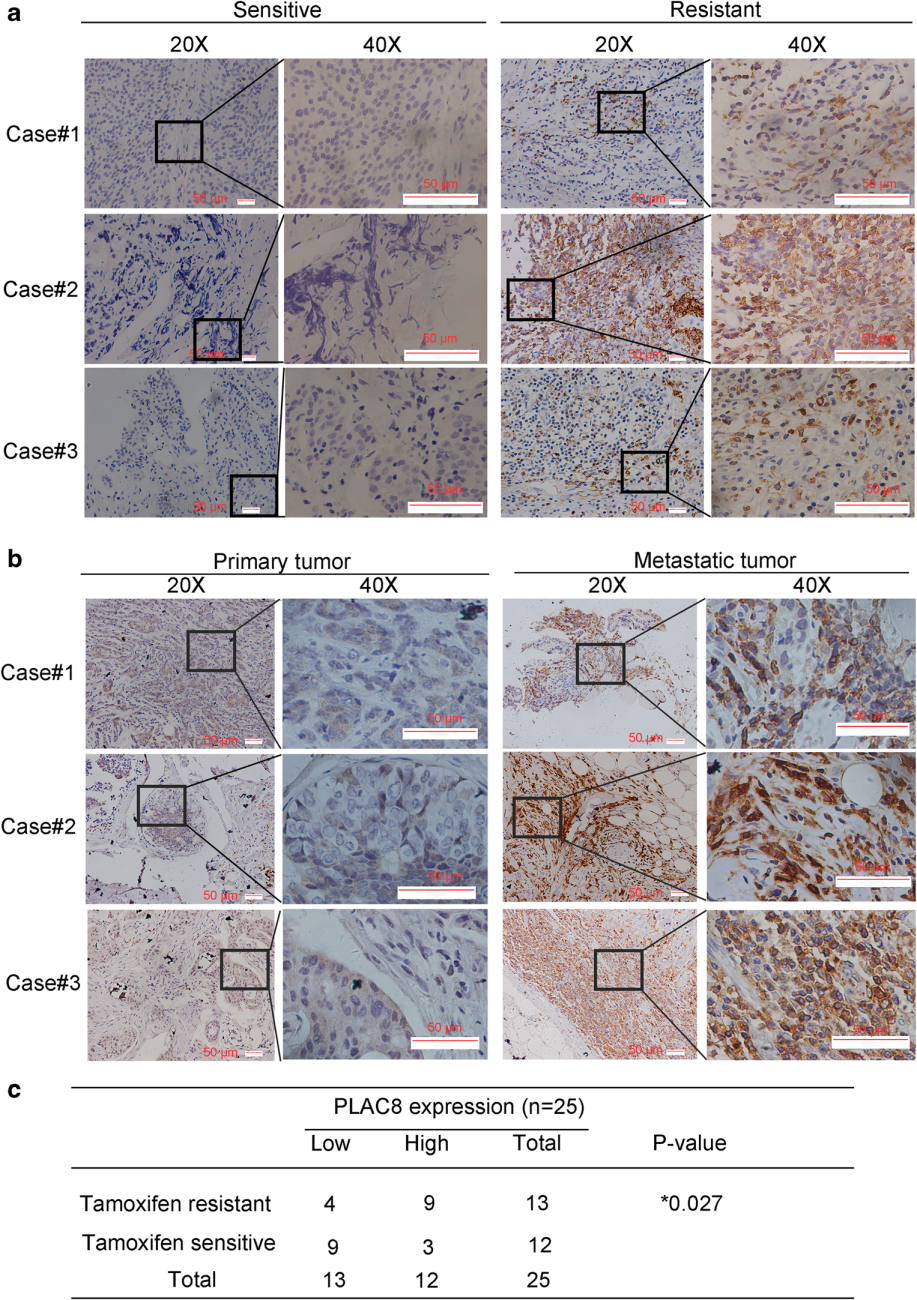
PLAC8 was associated with tamoxifen resistance in breast cancer patients. **a** Immunohistochemical analysis of PLAC8 expression in tamoxifen-resistant patients and tamoxifen-sensitive patients. **b** Immunohistochemical analysis of PLAC8 expression for primary tumor and their metastatic tumor from breast cancer patients. Scale bar represents 50μm. **c** The relationship between PLAC8 expression with tamoxifen resistance and sensitivity in primary breast cancer. PLAC8 expression was obtained for the intensity of PLAC8-positive staining (Low, 0-1; High 2-3). All data represent mean ± SD. **p < 0.01; *p < 0.05; NS, not significant

**Fig. 2:**
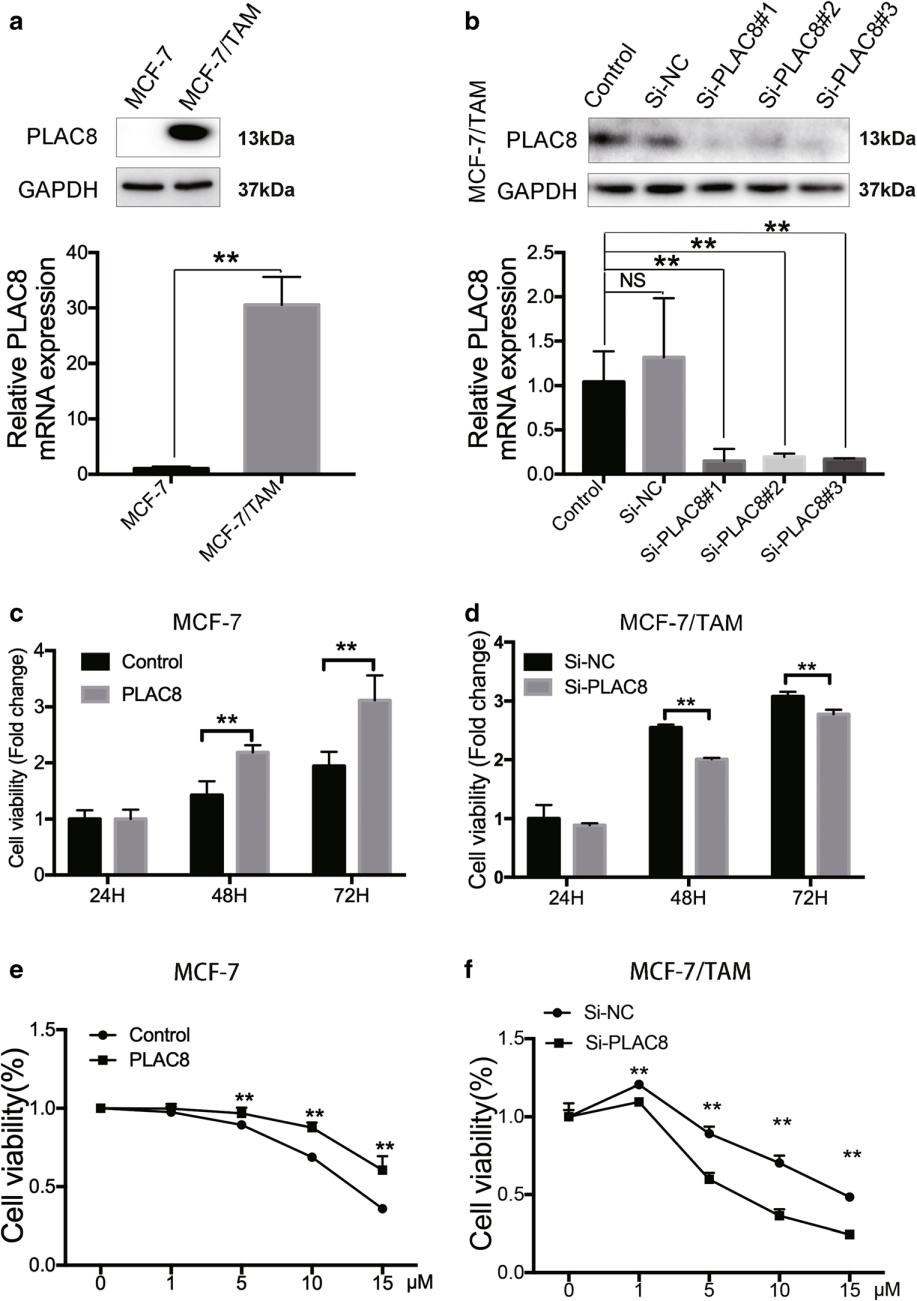
PLAC8 regulates MCF-7/TAM and MCF-7 cells proliferation. **a** The protein (upper) and mRNA levels (bottom) were relatively higher in MCF-7/TAM cells than in MCF-7 wild type. **b** MCF-7/TAM was transfected with PLAC8 siRNA; the interference effect of siRNA was determined by Western blot (upper) at 72 h after transfection and RT-PCR (bottom) at 48 h after transfection. **c** and **d** MTT assay was used to determine the cell viability of PLAC8 overexpressing cells or siRNA transfected cells. Control and Si-NC were chosen as the control. **e** and **f** Equal amount of MCF-7 cells or MCF-7/TAM cells under different PLAC8 condition was cultured with the various concentrations of tamoxifen for 48 h. Cell viability was determined by MTT assay.All data represent mean ± SD of three experiments performed in triplicate. **p < 0.01; *p < 0.05; NS, not significant

It has been identified that PLAC8 is related with cell division, differentiation, and apoptosis through different mechanisms [9, 21, 22]. Our previous study proved that PLAC8 was involved in the breast cancer progression [11]. This results trigger our interests about the involvement of PLAC8 in tamoxifen resistance. In this study, we further determined whether the feedback increase of PLAC8 might be associated with the response to tamoxifen in breast cancer. We first detected the PLAC8 expression of 12 samples of tamoxifen-sensitive and 13 samples of tamoxifen-resistant breast cancer tissues (Table s1). Among these patients, the expression of PLAC8 in tamoxifen resistant patients was higher compared with that in patients who were sensitive to tamoxifen(Fig. 1a and c). We also found that examined PLAC8 expression in the tissues of primary tissue and metastatic patients tumor, who were diagnosed with distant metastasis after receiving adjuvant tamoxifen treatment, PLAC8 expression was higher in metastatic tumor compared with their primary tissues(Fig. 1b and c). It indicated that the expression of PLAC8 might provide the information about tamoxifen resistant. Then, we established MCF-7/TAM by the prolonged growth of MCF-7 cells in 1 μM tamoxifen media over six months. As shown in Fig S1A and B , MCF-7/TAM was more resistant to tamoxifen than MCF-7 cells. The IC50 of tamoxifen in MCF-7 and MCF-7/TAM is 13.57 μM and 29.91 μM, respectively (Fig S1B). In addition, the morphology between MCF-7/TAM and MCF-7 wild type differed (Fig S1C and D). To determine the biological function of PLAC8 in MCF-7/TAM, we compared the protein and mRNA levels of PLAC8 between MCF-7/TAM and MCF-7 wild type. As shown in Fig. 2a, the protein and mRNA level of PLAC8 were significantly high in MCF-7/TAM. A reciprocal PLAC8 alteration was associated with responsiveness to tamoxifen, thereby suggesting that PLAC8 upregulation might contribute to tamoxifen resistance.

### Downregulation of PLAC8 can induce MCF-7/TAM sensitivity of tamoxifen via MAPK pathway

**Fig. 3:**
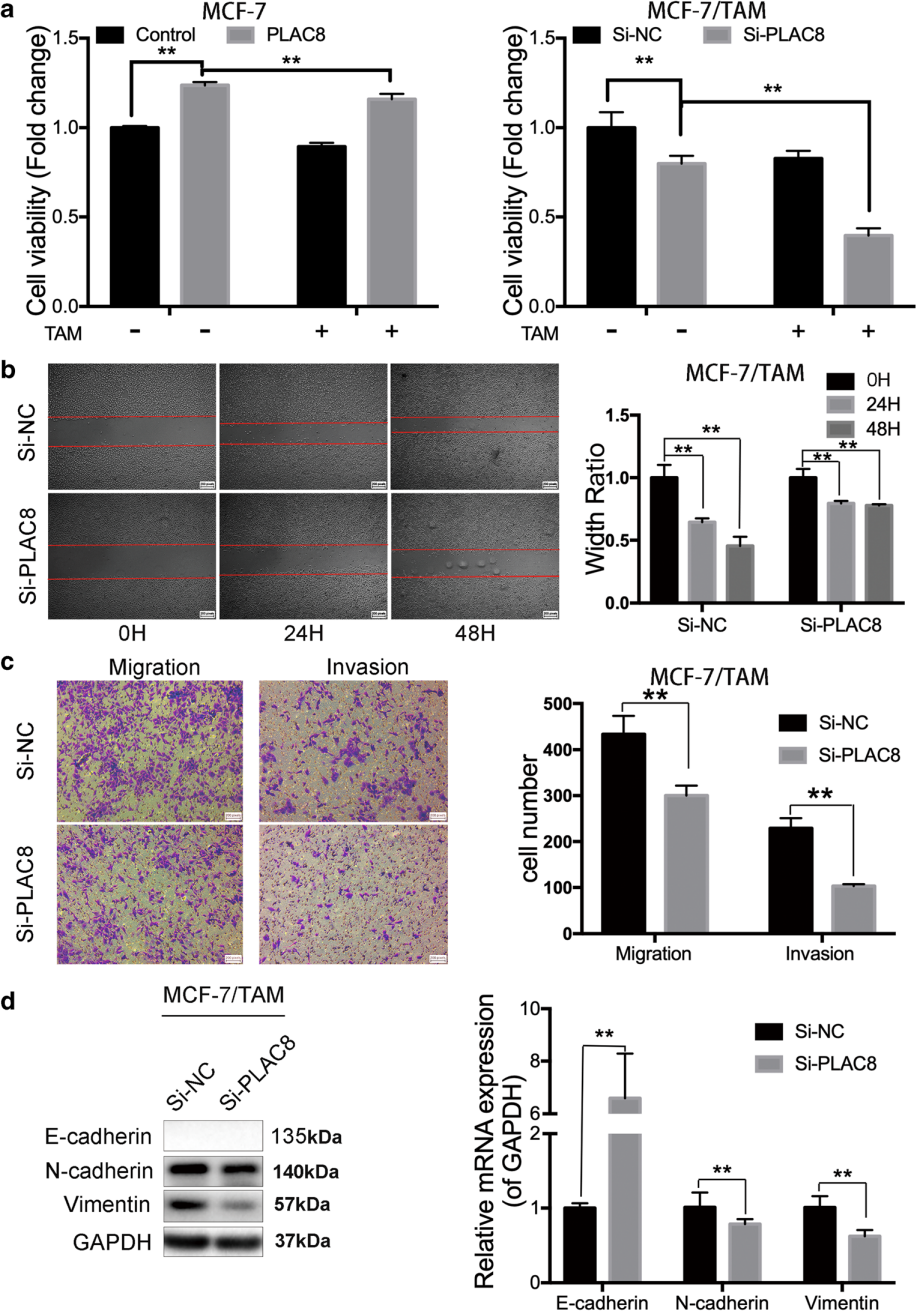
PLAC8 promotes MCF-7/TAM tamoxifen resistance and metastatic ability. **a** MTT assay showed that the cell viability of MCF-7 cells which was treated with tamoxifen (5 μM) or DMSO 24 h. Overexpressing PLAC8 MCF-7 cells grew faster than control cells. And MCF-7/TAM cells transfected with PLAC8 siRNA were relatively sensitive to tamoxifen (10 μM) than Si-NC cells. **b** Wound-healing assay was conducted in MCF-7/TAM that was transfected with empty or PLAC8 siRNA by a 200-μl pipette tip. Migration distance was measured at 0, 24, and 48 h after cells were scratched, the start time was chosen as the control (magnification 40×). **c** Representative images revealed the cell number of migration and invasion decreased in PLAC8 knock-down cells (magnification 100×). **d** Western blot (left) and RT-PCR (right) were carried out to determine the relative expression level of EMT-related markers. The expression was quantified and normalized to GAPDH. All data represent mean ± SD of three experiments performed in triplicate. **p < 0.01; *p < 0.05; NS, not significant

**Fig. 4:**
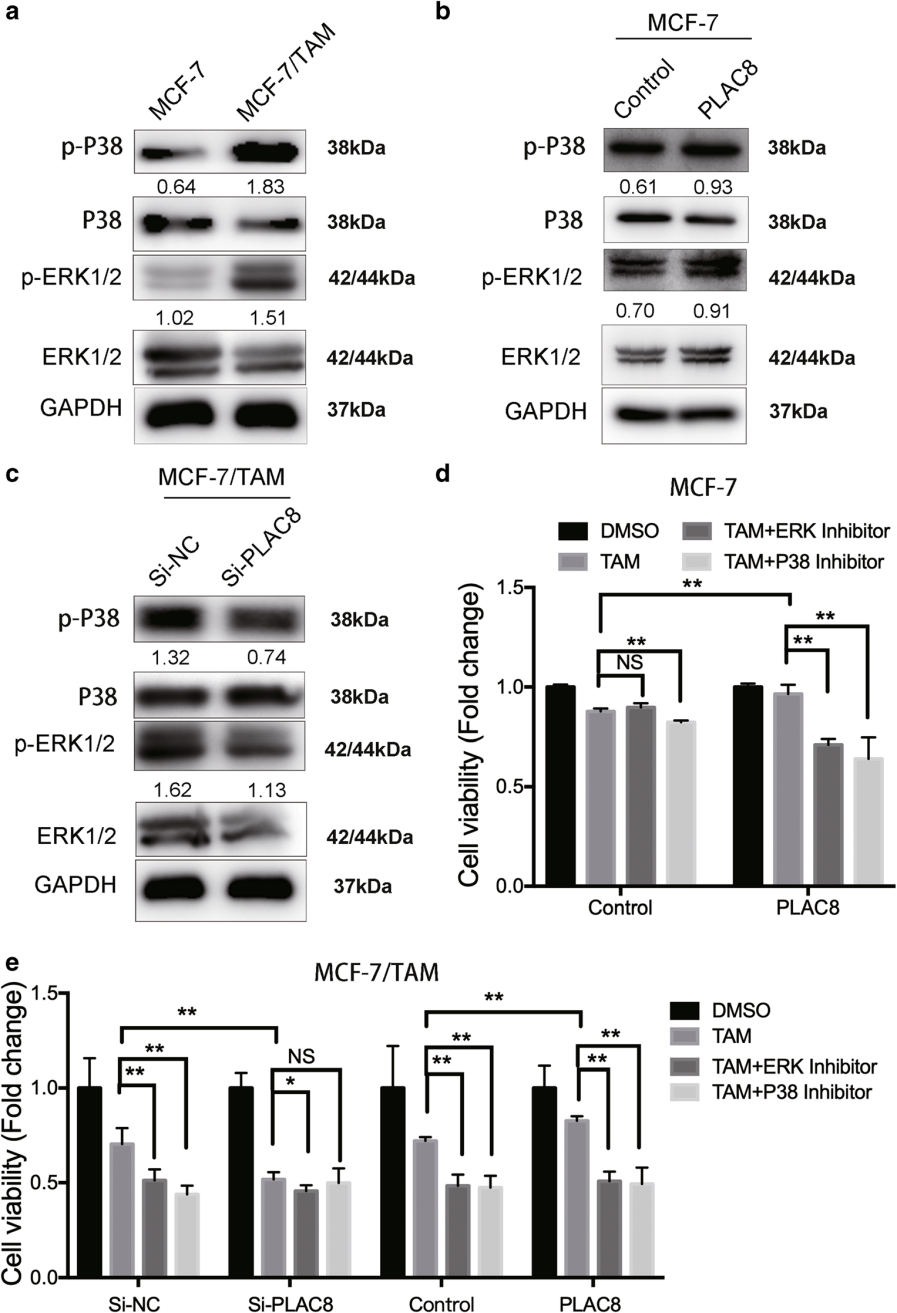
PLAC8 induces the activity of the MAPK pathway thus involving tamoxifen sensitivity. **a** Western blot was carried out to determine the relative expression level of P38, p-P38, ERK1/2, and p-ERK1/2. MCF-7/TAM had a relatively higher activity of p-P38 and p-ERK1/2 than MCF-7. **b** PLAC8 induced p-P38 and p-ERK1/2 protein expression in MCF-7 cells. However, knockdown of PLAC8 in MCF-7/TAM p-P38 and p-ERK1/2 was significantly reduced, as shown in (**c**). The expression was quantified and normalized to ERK, P38, and JNK by using Image J. **d** MCF-7 cells under different PLAC8 condition treated with tamoxifen (5 μM) alone or combined with MAPKs inhibitors(SCH772984 10 μM or SB2020190 20 μM) 24 h. Then cell viability was determined by MTT assay. **e** MCF-7/TAM transfected with PLAC8 siRNA or PLAC8 plasmid and subsequent treatment with tamoxifen (10 μM) alone or combined with MAPK inhibitors (SCH772984 10μM or SB2020190 20μM). All data represent mean ± SD of three experiments performed in triplicate. **p < 0.01; *p < 0.05; NS, not significant

PLAC8 upregulation might be correlated with tamoxifen resistance. To further confirm this conclusion, we reduced the PLAC8 expression in MCF-7/TAM cells by using siRNA and overexpressed PLAC8 level by plasmid in MCF-7 (Fig. 2b and Fig S1E). We used the Si-PLAC8#2 in our latter experiment. The changes in cell proliferation and migration were analyzed by MTT, wound-healing, and Transwell assays. MTT assay results showed that PLAC8 could induce cell proliferation both in MCF-7 and MCF-7/TAM (Fig. 2c and d). Then, we sought to detect whether PLAC8 could regulate tamoxifen sensitivity. Reducing the expression of PLAC8 induced the sensitivity of tamoxifen in MCF-7/TAM whereas overexpressing the level of PLAC8 protein promoted MCF-7 to be resistant to tamoxifen (Fig. 2e and f). Tamoxifen significantly reduced cell viability of the control vector group compared with PLAC8-overexpressed group in MCF7 cells (Fig. 3a). We also observed that silencing PLAC8 in MCF-7/TAM would significantly increase tamoxifen sensitivity and tamoxifen futher inhibited Si-PLAC8 group cells compared with Si-NC group cells (Fig. 3a).These results determined that PLAC8 could not only promote cell proliferation, but also induce tamoxifen resistance. Wound-healing and Transwell assay also showed that PLAC8 knockdown could suppress MCF-7/TAM migration and invasion (Fig. 3b and c). The related markers were determined by Western blot analysis and RT-PCR. Knock downing the PLAC8 repressed that the mesenchymal markers which included N-cadherin and vimentin (Fig. 3d). These results indicated that PLAC8 could promote MCF-7/TAM cell metastasis. MAPK pathway is a major component of signaling pathways involved in the regulation of cell proliferation, migration, and drug resistance [23, 24]. To determine the signaling molecules involved in tamoxifen resistance, we detected the protein levels of phosphorylated P38 (p-P38), P38, phosphorylated ERK1/2 (p-ERK1/2), and ERK1/2 by Western blot analysis in MCF-7 and MCF-7/TAM. As expected, the protein levels of p-P38 and p-ERK1/2 in MCF-7/TAM were higher than those in MCF-7 cells (Fig. 4a). Moreover, the levels of p-P38 and p-ERK1/2 were also downregulated in the knockdown of PLAC8 of MCF-7/TAM and upregulated in the overexpression of PLAC8 of MCF-7 (Fig. 4b and c). We further combined tamoxifen with MAPK inhibitors (including the ERK inhibitor and P38 inhibitor) in MCF-7 cells and found that cell viability was reduced significantly in the PLAC8 overexpressed group(Fig. 4d). In addition, the synergistic cyto-toxicity by tamoxifen and the MAPK inhibitors decreased when PLAC8 was knocked down in MCF-7/TAM cells(Fig. 4e). These data suggested that PLAC8 regulate tamoxifen resistance though activating the MAPK/ERK pathway.

### Ubiquitination of PLAC8 regulates the sensitivity of tamoxifen in MCF-7/TAM by curcrumin

**Fig. 5:**
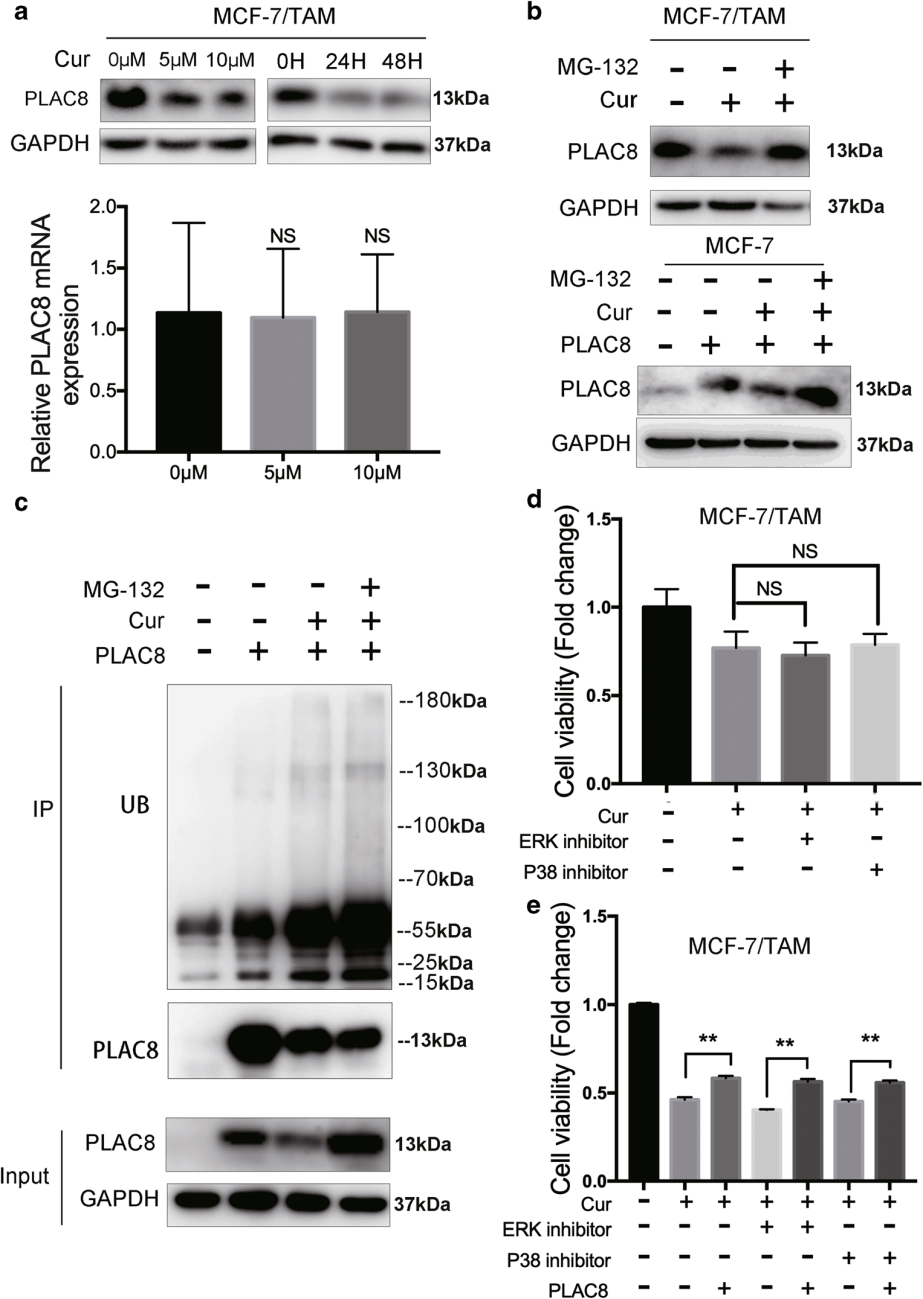
Relationship between the ubiquitin of PLAC8 and curcumin. **a** Western blot (upper) tested the expression level of PLAC8 decreased by curcumin in a dose- and time-dependent manner. RT-PCR (bottom) showed the mRNA expression level of PLAC8 was not changed by curcumin. The expression was quantified and normalized to GAPDH. **b** Western blot tested the expression level of PLAC8 decreased by treatment of curcumin 24 h (5 μM) could be recused by treatment of MG-132 6 h (10μM) in MCF-7/TAM (upper) and MCF-7 (bottom). **c** MCF-7/TAM cells were transfected with PLAC8. At 24 h post transfection, cells were treated with or without curcumin (5 μM). After 18 h later, cells were treated with or without MG132 (10 μM) for 6 h and immunoprecipitated with PLAC8 antibody. **d** Cell proliferation assay showed that curcumin (10 μM) alone or combined with MAPKs inhibitors (SCH772984 10 μM or SB2020190 20 μM) in MCF-7/TAM 24hr. **e** MCF-7/TAM treated with curcumin (10 μM) alone or combined with MAPKs inhibitors(SCH772984 10 μM or SB2020190 20 μM) and then overexpressing PLAC8 expression. All data represent mean ± SD of three experiments performed in triplicate. **p < 0.01; *p < 0.05; NS, not significant

Curcumin can inhibit many types of cancer, including breast cancer. Interestingly, the rapid decrease in the protein level of the PLAC8 could be induced by different concentrations and duration of curcumin treatment in MCF-7/TAM cell lines. Furthermore, although the PLAC8 protein level was significantly downregulated by curcumin, we observed no significant change in the amount of mRNA after curcumin treatment (Fig. 5a). Ubiquitination is a common post-translational modification of proteins. We next tested whether PLAC8 was regulated by this modification. As shown in Fig. 5b, the decrease in PLAC8 protein levels was significantly rescued by treating cells with MG-132, a reversible proteasome inhibitor both in MCF-7/TAM (upper) and MCF-7 (bottom). Endogenous PLAC8 was further immunoprecipitated and probed for ubiquitin. A significant accumulation of ubiquitylated PLAC8 was observed following curcumin treatment compared with the untreated control (Fig. 5c). Therefore, curcumin decreased the level of PLAC8 by increasing the ubiquitination of PLAC8 through the proteasome pathway. As expected, MAPK inhibitors could not further inhibit cell viability in MCF-7/TAM since curcumin reduced MAPK pathway activity through attenuating PLAC8 protein (Fig. 5d). Besides, increasing the PLAC8 protein could rescue cell viability that was inhibited by curcumin with or without MAPK inhibitors(Fig. 5e). The inhibition of PLAC8 expression and MAPK pathway by curcumin enhanced breast cancer cell sensitivity to tamoxifen and suggested that curcumin could be served as a effective drug for tamoxifen resistant breast cancer.

### Curcumin can suppress MCF-7/TAM cell proliferation, migration, and invasion

**Fig. 6:**
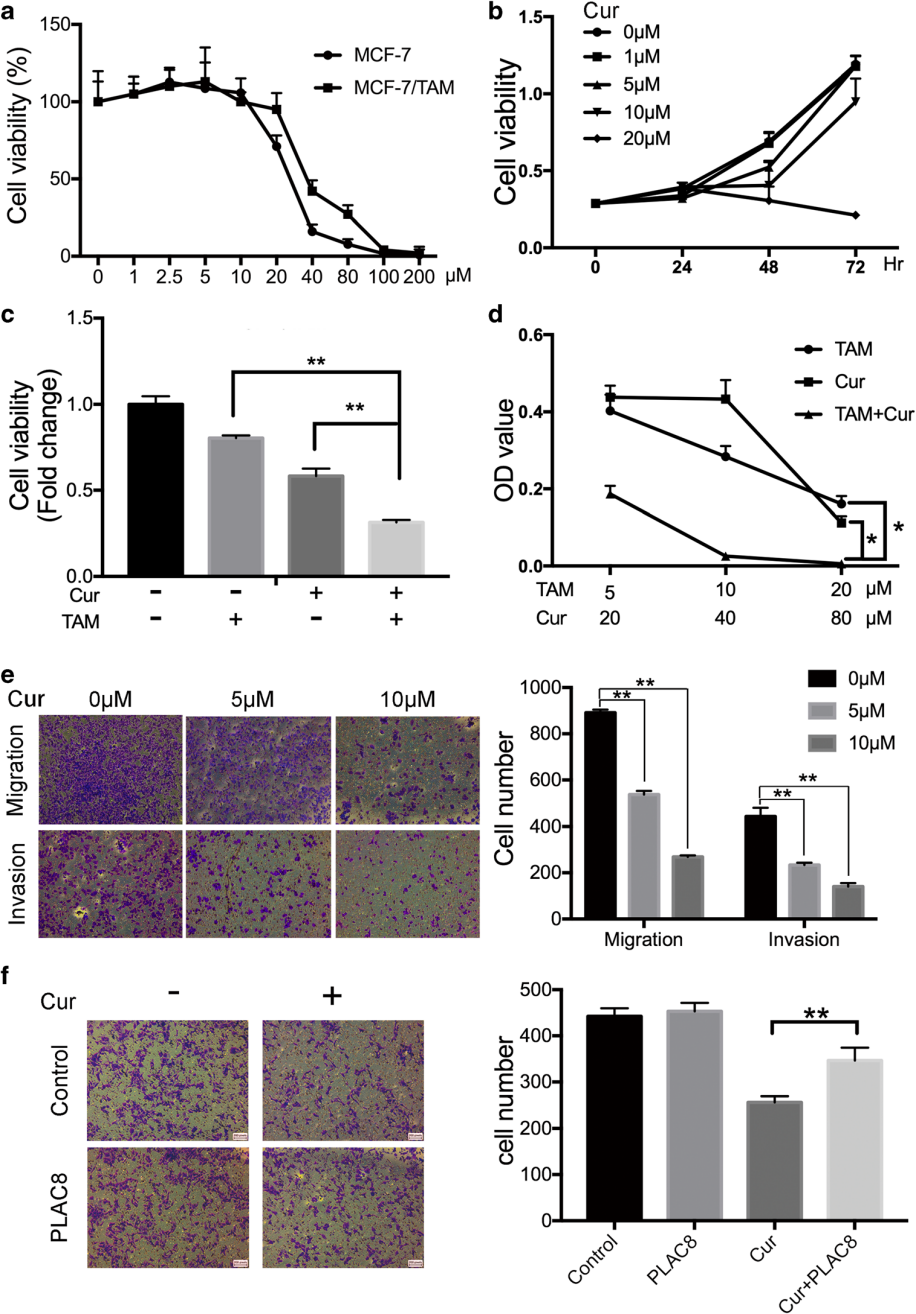
Curcumin reduces the ability of proliferation, migration and invasion in the MCF-7/TAM. **a** MCF-7 and MCF-7/TAM cells were cultured with various concentrations of curcumin for 48 h. Cell viability rate was determined by MTT assays. **b** MCF-7/TAM was treated with different concentrations of curcumin for 0, 24, 48, and 72 h. **c** Cell viability assay showed curcumin combined with tamoxifen significantly inhibited MCF-7/TAM proliferation compared with curcumin (10 μM) alone or tamocxifen(10 μM). **d** MTT assay showed that the combination of tamoxifen and curcumin significantly inhibited MCF-7/TAM ability in a dose-dependent manner. **e** Representative images revealed the cell number of migrating and invading decreased in a dose-dependent manner (magnification 100×). **f** Transwell migration assay determined that the overexpression of PLCA8 reversed the reduced cell number caused by curcumin (5 μM). All data represent mean ± SD of three experiments performed in triplicate. **p < 0.01; *p < 0.05; NS, not significant

According to the above results, the IC50 values after treatment with curcumin for 48 h were 25.4 μM in MCF-7 cells and 30.2 μM in MCF-7/TAM cells (Fig. 6a). MCF-7/TAM was treated with increasing curcumin concentrations for different durations, and cell proliferation was analyzed. The proliferation was inhibited in a dose- and time-dependent manner according to the MTT, especially treated with curcumin after 48 h (Fig. 6b). The long-term effects of curcumin on MCF-7/TAM cells proliferation were investigated using colony formation assay (Fig S2A), which was consistent with the results of MTT. In addition, a combination of tamoxifen and curcumin inhibited MCF-7/TAM proliferation more than either of the agents alone in a dose-dependent manner (Fig. 6c and d). As shown in Fig. 6e and Fig S2B, curcumin attenuated the migration and invasion capability of MCF-7/TAM cells in a dose-dependent manner. The change in the mRNA and protein marker of the epithelial-mesenchymal transition was consistent with the outcome of the wound-healing and Transwell assay (Fig S2C). Moreover, transwell assay showed that the reduction in cell migration and invasion caused by curcumin was reversed by overexpression of PLAC8 (Fig. 6f). Collectively, curcumin could inhibit breast cancer cell proliferation, migration, invasion, and reverse tamoxifen resistance through affecting PLAC8 protein stability.

### The combination of tamoxifen and curcumin significantly inhibited tumor growth in vivo

**Fig. 7:**
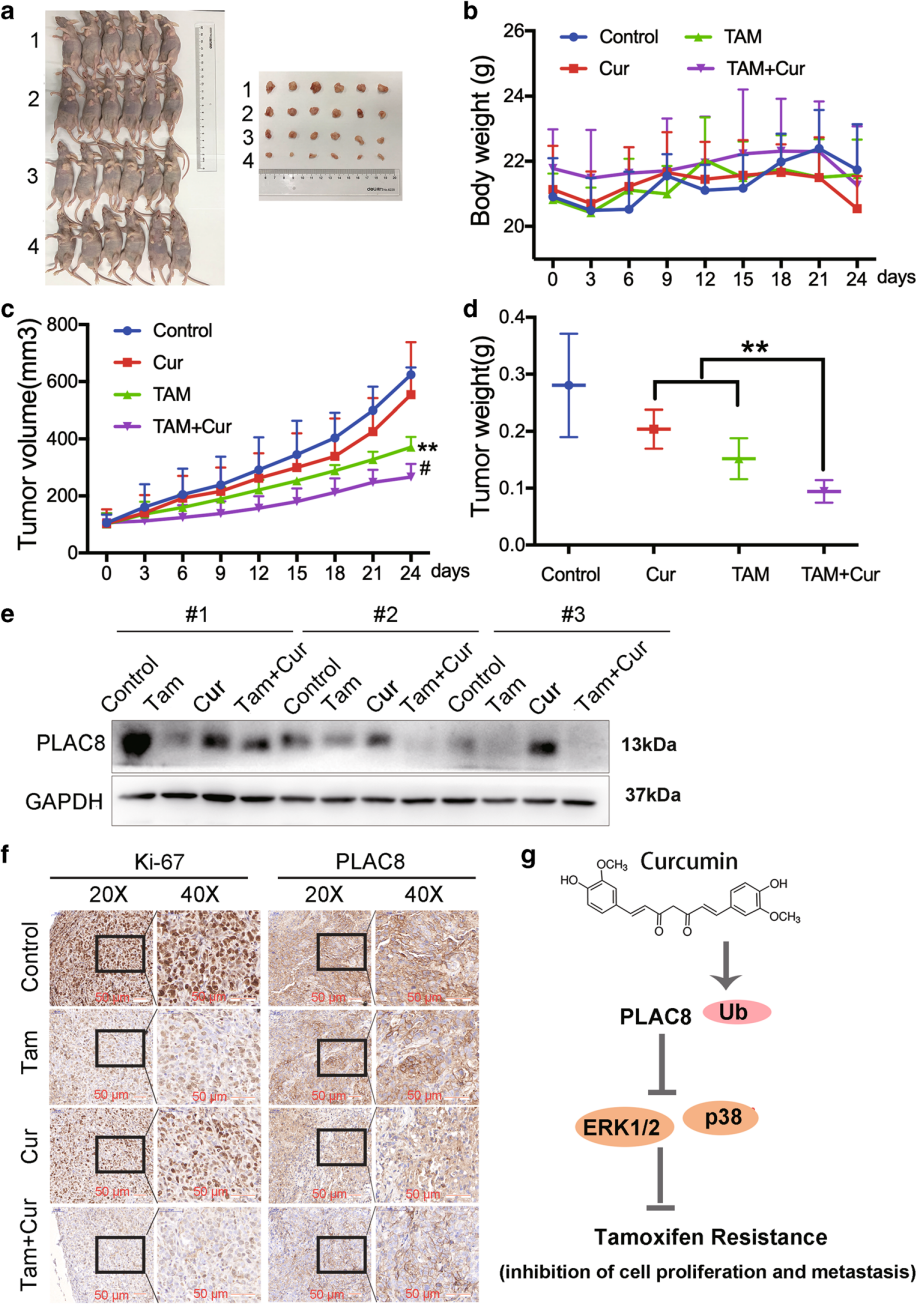
Tamoxifen inhibited tumor growth and showed a synergistic activity with curcumin in vivo. (**a**) Body weight (**b**), tumor volume (every 3 days) (**c**) and tumor weight (day 24 after treating drug) (**d**) of xenograft-bearing mice in four groups. 1, Control; 2, Curcumin; 3, Tamoxifen; 4, Curcumin and Tamoxifen. (^∗*^P < 0.01 vs. control group [upper], ^#^*P* < 0.05 vs. single-treated group). **e** Western blot analysis results showed that curcumin induced the downregulation of PLAC8 protein levels in MCF-7/TAM xenograft-bearing mice. **f** IHC staining showed the expression of Ki67 and PLAC8. Scale bar represents 50 μm. **g** Hypothesis model of curcumin action on regulating BC progression. Our indicated that PLAC8 could induce the tamoxifen resistance, thus affecting the ERK1/2-p38 kinase pathways. In addition, curcumin decreased the level of PLAC8 through ubiquitination modification. This mechanism may explain the potential mechanism of tamoxifen resistance to breast cancer

Xenografts in nude mice were used to evaluate the antitumor effect of tamoxifen or curcumin treatment alone or in combination in vivo (Fig. 7a and b). Treatment with tamoxifen or curcumin alone inhibited MCF-7/TAM xenograft growth compared with the control. However, the combination of tamoxifen and curcumin inhibited tumor growth more than either of the agents alone with no major change in the body weight (*P* < 0.01, Fig. 7c and d). Moreover, curcumin treatment alone or the combination of tamoxifen and curcumin could decrease PLAC8 expression in the xenograft tumors (Fig. 7e). IHC analysis revealed that curcumin inhibited PLAC8 expression in the xenograft (Fig. 7f). Therefore, the combination of tamoxifen and curcumin significantly inhibited tumor growth in vivo and curcumin could be a promsing drug for tamoxifen-resistant patients.

## Discussion

Breast cancer is the most common tumor in women. Tamoxifen is widely recognized as the first-line drug for breast cancer patients who are estrogen receptor positive; however, the emergence of drug resistance represents a major setback that requires urgent attention [17, 25]. The mechanism of tamoxifen resistance and increasing sensitivity to tamoxifen, as well as the mutation of the target gene and the activation of the pathway, have been widely studied to improve the overall survival of patients with breast cancer. For example, the overexpression of the MACROD2 mediates estrogen-independent growth and tamoxifen resistance [26]. miRNAs regulate the hallmarks of tamoxifen resistance, including the regulation of the cell proliferation, cell death, apoptosis, invasion, and metastasis. miR-186-3p/EREG axis orchestrates tamoxifen resistance in ER-positive breast cancer [27]. The expression of miR-135a, which is partially dependent on the activation of the ERK1/2 and AKT pathways, was downregulated in ER-positive breast cancer cells with acquired tamoxifen resistance [28]. However, the accurate mechanism of tamoxifen resistance is unclear and should be further studied.

PLAC8 is a 115–amino acid, cysteine-rich protein [29]. PLAC8 can regulate the proliferation of several cancer types by affecting different targets and pathways. In hepatocellular carcinoma, PLAC8 is a tumor suppressor regulated by miR-185-5p, and it also suppresses cell proliferation [30]. However, PLAC8 promotes the carcinogenesis and EMT of nasopharyngeal carcinoma cells via the TGF-β/Smad pathway [31]. PLAC8 positively regulates trophoblast invasion and migration by upregulating the activation of Rac1 and Cdc42 [9]. PLAC8 can promote BC prolifetion as we previously discussed. In this study, we aimed to identify the biological roles of PLAC8 in tamoxifen resistance.

Curcumin is one of the most extensively studied natural products in the past, and it has been implicated in various diseases. Moreover, curcumin can regulate the progression of various cancer types in vitro and in vivo by regulating different genes. It indicates that curcumin has remarkable anti-tumor effects including in breast cancer. Researches have reported that curcumin reduces chemotherapy resistance in breast cancer stem cells by regulating Bcl-2 family-mediated apoptosis [32]. In addition, curcumin impedes 26S proteasome activity via DYRK2 inhibition, and its treatment significantly reduced tumor volume in a TNBC mouse xenograft model [33].

In this study, we used a tamoxifen-resistant cell line and xenograft models to examine whether PLAC8 inhibition increased sensitivity to tamoxifen and to reveal the underlying mechanisms of curcumin and PLAC8.

Our results showed that PLAC8 could increase tamoxifen resistance and promote the metastasis of MCF-7/TAM cells by increasing the expressions of the N-cadherin and vimentin. Moreover, the knockdown of the PLAC8 decreased cell ability and induced drug sensitivity by inactivating the MAPK/ERK pathway. In addition, curcumin could significantly reduce cancer proliferation in vivo in a time- and dose-dependent manner. Curcumin also increased the protein and mRNA expressions of E-cadherin and decreased the protein and mRNA expressions of the N-cadherin and vimentin, thereby regulating the migration and invasion of MCF-7/TAM cells which was consistent with knocking down PLAC8 in MCF-7/TAM cells. Ubiquitin-coupled degradation of numerous junctional proteins is critical in epithelial de-differentiation and the acquisition of a motile and invasive phenotype. Pharmacological interventions targeting the ubiquitin-proteasome pathway could affect carcinoma cell invasion and metastasis. For example, TIAM1 could ubiquitylate the HUWE1; Peli1 mediates the formation of K63-linked ubiquitination of NBS1; and OTUB1 deubiquitinase affects SLC7A11 protein [34–36]. We determined that curcumin could induce the ubiquitin of PLAC8, thereby degrading the PLAC8 protein. Moreover, the overexpression of PLAC8 could partially reverse the effect of curcumin. In vivo, we concluded that curcumin combined with tamoxifen could significantly reduce tumor formation compared with only curcumin or tamoxifen. Our results clarified the underlying relationship between the PLAC8 and curcumin in BC.To the best of our knowledge, we are the first to demonstrate that curcumin increases the ubiquitin of PLAC8 protein and augments endocrine-sensitivity to tamoxifen in breast cancer in vitro and in vivo.

We demonstrated that the knockdown of PLAC8 by the ubiquitin increased the sensitivity of tamoxifen in breast cancer cells. In addition, curcumin might reduce the level of the PLAC8 through the proteasome pathway (Fig. 7g). PLAC8 might be a novel target gene, and curcumin might be a potential adjuvant therapeutic agent for the treatment of tamoxifen-resistant patients.

## Supplementary information

Supplementary Figure 1 Characteristics of MCF-7/TAM cells. (A) Colony assay showed that tamoxifen suppressed colony formation in MCF-7 cells compared with MCF-7/TAM. Cells were treated with different concentrations tamoxifen and were allowed to form colonies in fresh medium for 14 days. The group of 0 μM was chosen as the control. (B) Cells were treated with tamoxifen at gradient concentrations for 48 hr, and IC50s were measured using MTT assay. (C) MCF-7/TAM cells exhibited an elongated, spindle-shaped morphology, whereas MCF-7 wild type cells exhibited a cuboidal shape (magnification, 100×). (D) Scanning electron microscope image of MCF-7 and MCF-7/TAM. (E) MCF-7 was transfected with PLAC8 or control vector plasmid. The interference effect was determined by western blot (left) 72 hr and RT-PCR (right) 48 hr after transfection. All data represent the mean ± SD of three experiments performed in triplicate. **p < 0.01; *p < 0.05; NS, not significant. (PNG 6377 kb)

High resolution image (TIF 10675 kb)

Supplementary Figure 2 (A) Colony assay showed that curcumin suppressed colony formation in MCF-7/TAM in a dose-dependent manner for 14 days. The group at 0 μM was chosen as the control. (B) Wound-healing assay was conducted in MCF-7/TAM cultured in various concentrations of curcumin. Migration distance was measured at 0, 24, and 48 hr after cells were scratched using a 20 μl pipette tip; the start time was chosen as the control (magnification 40×). (c) Western blot (left) and RT-PCR (right) tested the relative expression level of EMT-related markers in various concentrations of curcumin. All data represent mean ± SD of three experiments performed in triplicate. **p < 0.01; *p < 0.05; NS, not significant. (PNG 6377 kb)

High resolution image (TIF 4541 kb)

ESM 1 (DOCX 18 kb)
