# Supplementary figures and images for: Regulation of tamoxifen sensitivity by the PLAC8/MAPK pathway axis is antagonized by curcumin-induced protein stability change
Source: J Mol Med (Berl). 2021 Feb 21;99(6):845–58. doi: 10.1007/s00109-021-02047-5 (PMC8164584; doi:10.1007/s00109-021-02047-5)

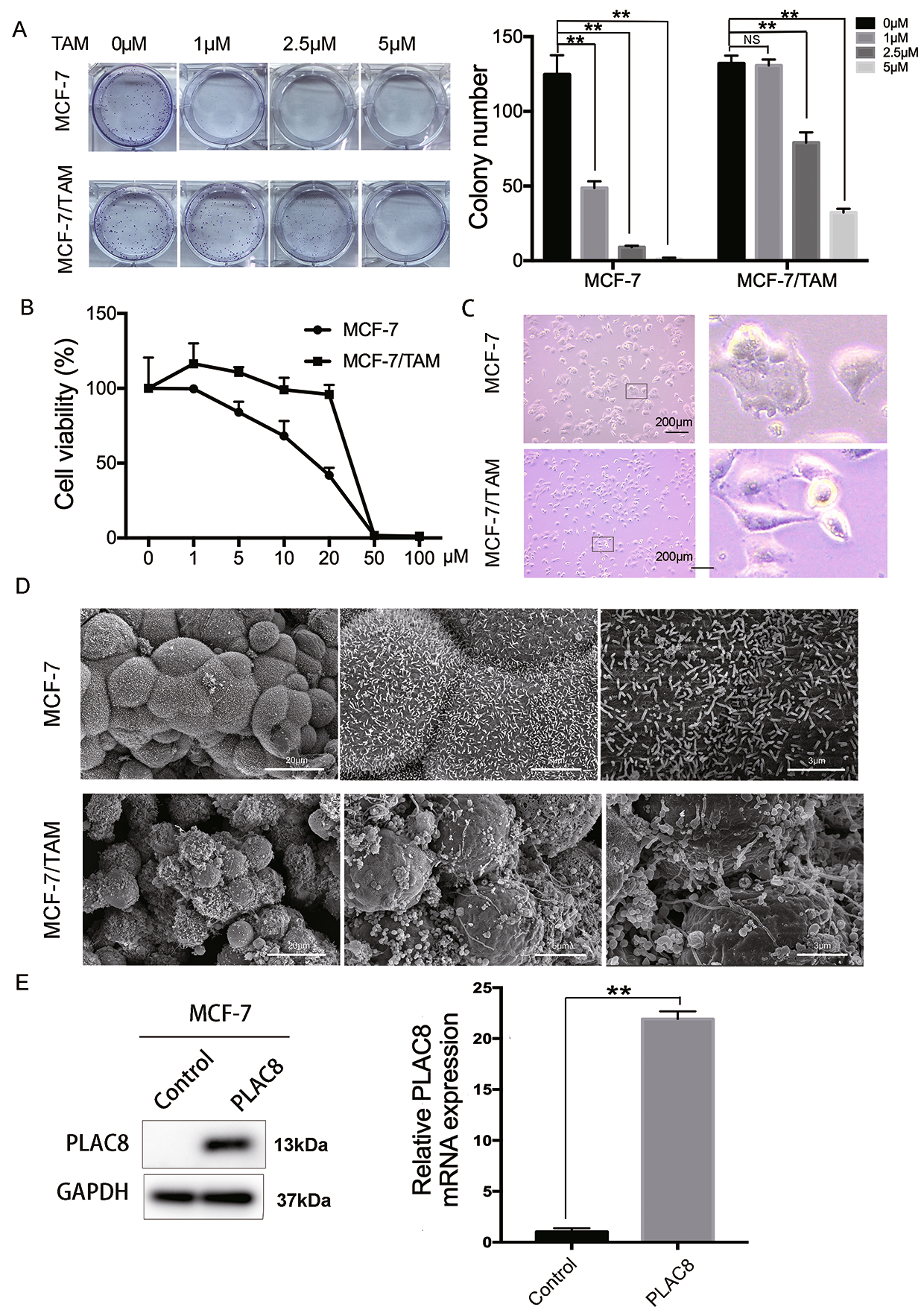

Supplement: Supplementary file 1 — Characteristics of MCF-7/TAM cells. (A) Colony assay showed that tamoxifen suppressed colony formation in MCF-7 cells compared with MCF-7/TAM. Cells were treated with different concentrations tamoxifen and were allowed to form colonies in fresh medium for 14 days. The group of 0 μM was chosen as the control. (B) Cells were treated with tamoxifen at gradient concentrations for 48 hr, and IC50s were measured using MTT assay. (C) MCF-7/TAM cells exhibited an elongated, spindle-shaped morphology, whereas MCF-7 wild type cells exhibited a cuboidal shape (magnification, 100×). (D) Scanning electron microscope image of MCF-7 and MCF-7/TAM. (E) MCF-7 was transfected with PLAC8 or control vector plasmid. The interference effect was determined by western blot (left) 72 hr and RT-PCR (right) 48 hr after transfection. All data represent the mean ± SD of three experiments performed in triplicate. **p < 0.01; *p < 0.05; NS, not significant. (PNG 6377 kb) [file 109_2021_2047_Fig8_ESM.png]

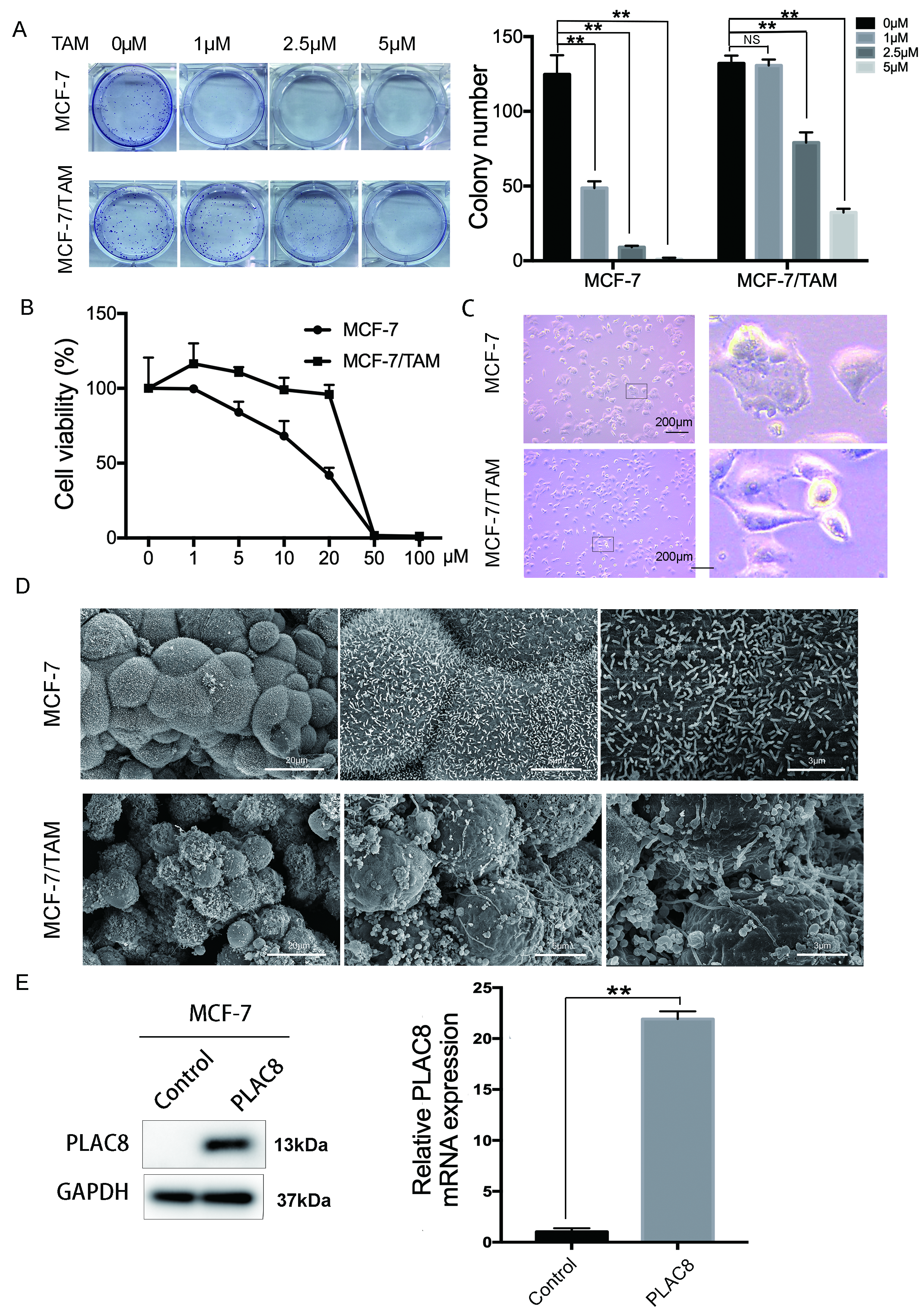

Supplement: Supplementary file 2 — High resolution image (TIF 10675 kb) [file 109_2021_2047_MOESM1_ESM.tif]

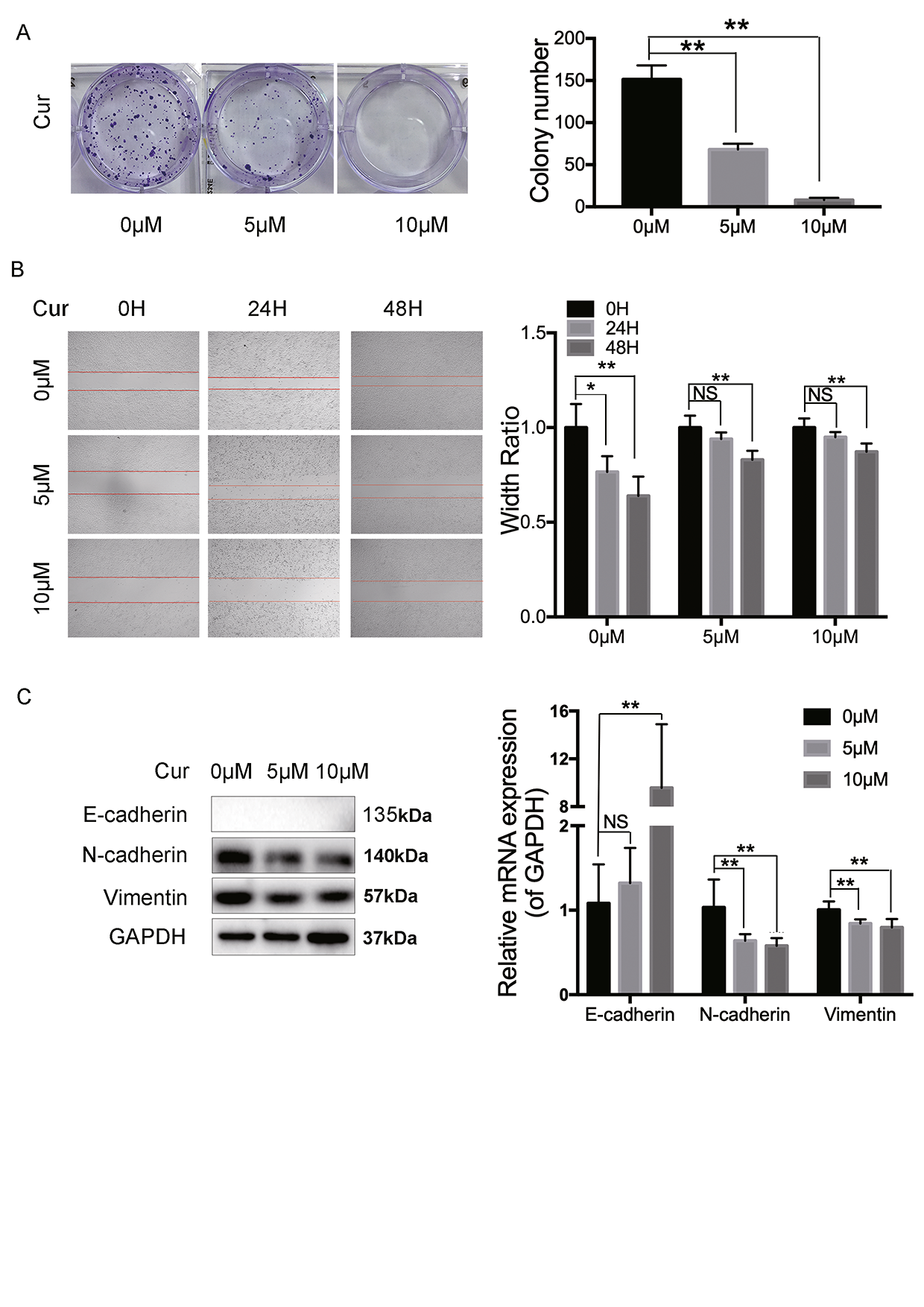

Supplement: Supplementary file 3 — (A) Colony assay showed that curcumin suppressed colony formation in MCF-7/TAM in a dose-dependent manner for 14 days. The group at 0 μM was chosen as the control. (B) Wound-healing assay was conducted in MCF-7/TAM cultured in various concentrations of curcumin. Migration distance was measured at 0, 24, and 48 hr after cells were scratched using a 20 μl pipette tip; the start time was chosen as the control (magnification 40×). (c) Western blot (left) and RT-PCR (right) tested the relative expression level of EMT-related markers in various concentrations of curcumin. All data represent mean ± SD of three experiments performed in triplicate. **p < 0.01; *p < 0.05; NS, not significant. (PNG 6377 kb) [file 109_2021_2047_Fig9_ESM.png]

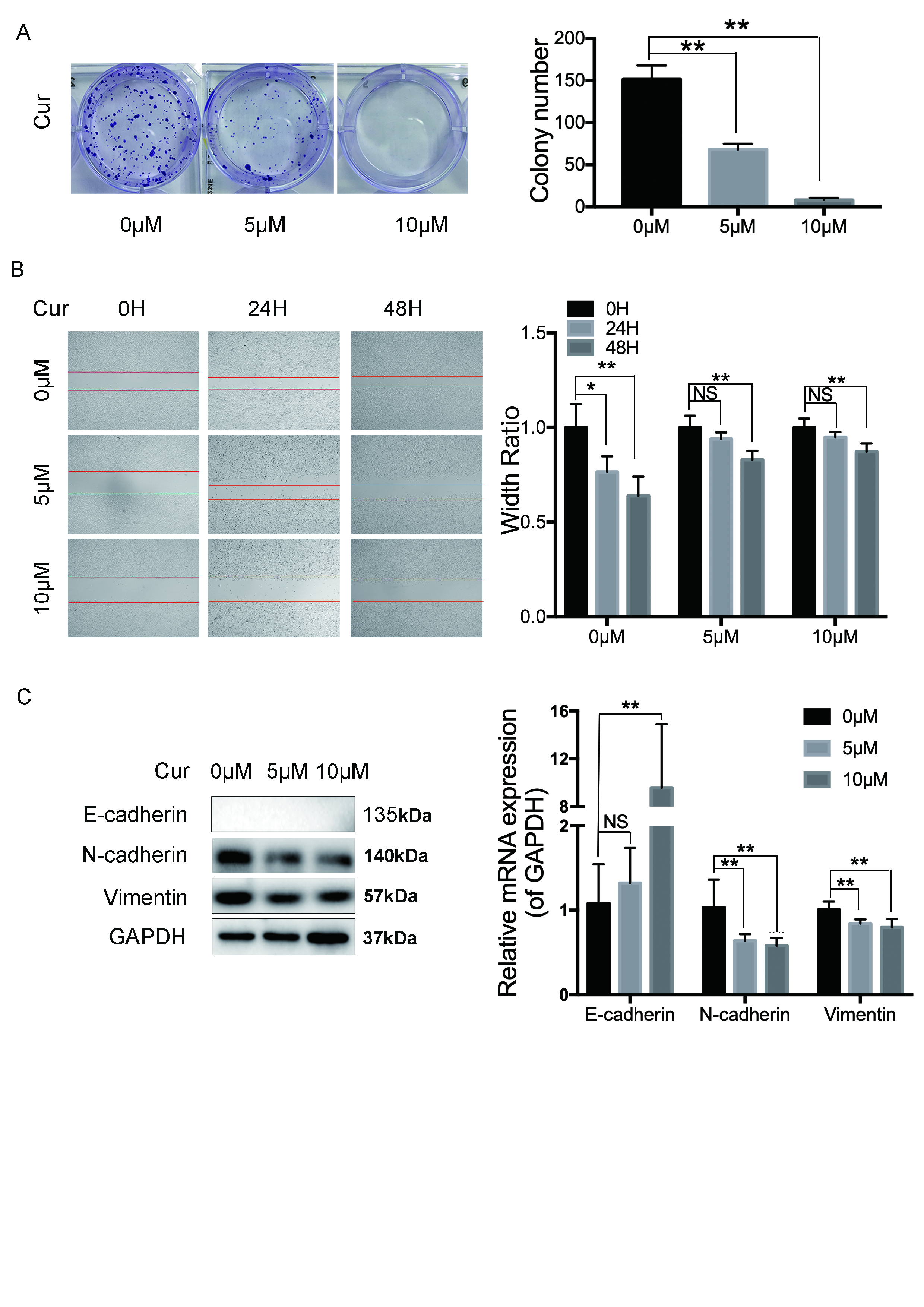

Supplement: Supplementary file 4 — High resolution image (TIF 4541 kb) [file 109_2021_2047_MOESM2_ESM.tif]
