# Supplementary material for: Regulation of tamoxifen sensitivity by the PLAC8/MAPK pathway axis is antagonized by curcumin-induced protein stability change
Source: J Mol Med (Berl). 2021 Feb 21;99(6):845–58. doi: 10.1007/s00109-021-02047-5 (PMC8164584; doi:10.1007/s00109-021-02047-5)
Supplement: Supplementary file 5 — (DOCX 18 kb) [file 109_2021_2047_MOESM3_ESM.docx]

Table S1

Supplementary information of 25 patients

| Case | Age | Duration of Tamoxifen treatment (years) | Metastasis | PLAC8 expression |
| --- | --- | --- | --- | --- |
| 1 | 36 | 4 | No | Low |
| 2 | 47 | 3 | No | Low |
| 3 | 41 | 4 | No | Low |
| 4 | 42 | 5 | No | Low |
| 5 | 46 | 4 | No | High |
| 6 | 70 | 4 | No | Low |
| 7 | 71 | 4 | No | Low |
| 8 | 55 | 5 | No | High |
| 9 | 48 | 5 | No | High |
| 10 | 48 | 4 | No | Low |
| 11 | 45 | 5 | No | Low |
| 12 | 52 | 5 | No | Low |
| 13 | 50 | 2 | Yes | High |
| 15 | 60 | 2 | Yes | High |
| 15 | 48 | 5 | Yes | High |
| 16 | 44 | 3 | Yes | High |
| 17 | 55 | 2.5 | Yes | High |
| 18 | 62 | 3 | Yes | Low |
| 19 | 40 | 2.5 | Yes | High |
| 20 | 42 | 2 | Yes | High |
| 21 | 65 | 4 | Yes | Low |
| 22 | 47 | 3 | Yes | Low |
| 23 | 64 | 3 | Yes | High |
| 24 | 63 | 4 | Yes | High |
| 25 | 48 | 3.5 | Yes | Low |
